# Action observation treatment may improve daily living activities and verb recovery in Parkinson’s disease-dementia: findings from a preliminary randomized controlled trial

**DOI:** 10.3389/fnagi.2024.1488881

**Published:** 2024-12-05

**Authors:** Lucia Paciaroni, Elena Mastrosanti, Leonardo Biscetti, Susy Paolini, Sara Mauri, Paolo Fabbietti, Giovanni Renato Riccardi, Marco Bruno Luigi Rocchi, Giuseppe Pelliccioni

**Affiliations:** ^1^Unit of Neurology, INRCA-IRCCS, National Institute of Health and Science on Aging, Ancona, Italy; ^2^Department of Biomolecular Sciences, University Carlo Bo, Urbino, Italy; ^3^Unit of Geriatric Pharmacoepidemiology, INRCA-IRCCS, National Institute of Health and Science on Aging, Ancona, Italy; ^4^Clinical Unit of Physical Rehabilitation, INRCA-IRCCS, National Institute of Health and Science on Aging, Ancona, Italy

**Keywords:** action observation treatment, Parkinson’s disease, dementia, daily living activities, verbs retrieval

## Abstract

**Background and objectives:**

Action observation treatment (AOT) is a novel rehabilitation approach aimed to the recovery of both motor and linguistic deficits in subjects with brain lesions. The aim of the present randomized controlled study was to assess the benefits of AOT treatment in the activities of daily living (ADLs) and in the linguistic abilities of the patients with Parkinson’s disease dementia (PDD) at mild–moderate stage (Hoehn & Yahr’s stage scale: 2–3).

**Methods:**

Twenty patients were enrolled and randomly assigned to an experimental group (submitted to AOT) or to a control group. The experimental group (AOT-group) underwent the vision of a video containing 6 complex ADLs, while the control group (C-group) was subjected to a video-clip regarding semantic information of a geographical-naturalistic type without motor content. The treatment duration was 4 weeks. All patients underwent assessment before and after the treatment by the following tools: Unified Parkinson’s Disease Rating Scale Part III (UPDRS-Part III), Alzheimer’s Disease Cooperative Study-Activities of Daily Living Scale (ADCS-ADL), Direct Assessment of Functional Status (DAFS) and subtest Verb Naming of Analysis of Aphasic Deficit Battery (BADA). Paired samples *t* test was performed to compare all the variables of interest in the time, dividing by groups. *p*-value<0.05 was considered significant in all analyses.

**Results:**

AOT-group showed an improvement from baseline to the end of study in ADCS-ADL (*p* = 0.001), BADA (*p* = 0.011) and DAFS (*p* = 0.005), while C-group did not change significantly in the time.

**Conclusion:**

These preliminary results suggest the potential efficacy of AOT in rehabilitation of ADLs and verb retrieval in people with PD. Further studies will be necessary to verify these findings.

## 1 Introduction

Parkinson’s disease (PD) is the second most common neurodegenerative disorder after Alzheimer’s disease, thus representing one of the chronic diseases with the major medical, psychological and economic burden.

The cognitive disturbances are frequently found in PD patients, sometimes already at the time of diagnosis and during the early stages of disease. These include a mild cognitive impairment (MCI) found at onset in approximately 26.7% of patients (Litvan et al., 2012), up to an established diagnosis of dementia (Emre et al., 2007). It has been estimated that at least 75% of PD patients will meet dementia criteria 10 years after the initial diagnosis (Aarsland and Kurz, 2010).

In addition to early pharmacological treatment of PD (van den Heuvel et al., 2021), cognitive stimulation training in the early stage of the disease has been proposed to improve cognitive function.

The methods can be divided into traditional cognitive training (paper –pencil tasks training), computerized cognitive training and training with applied virtual reality technology to improve the accessibility of cognitive training (Angelucci et al., 2015; Hindle et al., 2016; Kalron and Zeilig, 2015; Leung et al., 2015; Petrelli et al., 2014, 2015; Yu and Wu, 2022).

The rehabilitation program should be adapted to the different stages of the disease and to the transition of each state. Few studies have investigated cognitive training in patients with PD-dementia complex (PDD), when difficulties in performing the daily living activities (ADLs) arise (Sperens et al., 2020).

Recent research in the neuro-rehabilitation field has reported the potential efficacy of a new strategy, the Action Observation Treatment (AOT) in the recovery of impaired motor abilities for a vast number of clinical conditions, spanning from traumatological patients to brain injuries and neurodegenerative diseases (Rizzolatti et al., 2021).

This technique is an approach based on the paradigm of mirror neurons (Bonini et al., 2022), meaning that the observation of motor actions performed by other individuals activates the motor system of those who observe in a similar manner to the activation that occurs during the actual execution of the movement. There is indeed an overlap between the brain areas that are active during the observation of an action and the ones active during the execution (Molenberghs et al., 2012).

Moreover, there is evidence that the observation of actions conducted by others improves lexical recovery and can be used in the rehabilitation of aphasia to recover naming of verbs (Marangolo et al., 2012; Bonifazi et al., 2013).

A future application of AOT could be training interventions focused not only to a single motor act, disregarding their concatenation into a goal-directed action, but also to the whole chain of movements composing a complex action (Rizzolatti et al., 2021).

There are some specific studies in PD patients on the effects of neuro-cognitive rehabilitation (including AOT) based on mirror neurons paradigm (Abbruzzese et al., 2015; Buccino, 2014; Monticone et al., 2015; Poliakoff, 2013; Wang et al., 2016).

Pelosin et al. (2010, 2018) reported that the AOT may be proposed as an innovative and effective method for improving gait disturbances and mobility, suggesting that this technique could represent a new rehabilitation approach. More specifically, it has been observed that subjects submitted to videos with active movements improved their freezing of gait more than patients who watched “static” videos. A further meaningful result has been obtained for the reduction of bradykinesia after only one session of AOT in PD patients compared to subjects submitted to acoustic cue (Pelosin et al., 2013).

A systematic review by Giannakopoulos et al. (2022) concluded that AOT improves functional ability and motor control in PD patients, with the intervention dose and the characteristics of the stimulus playing a decisive role in its efficacy.

Furthermore, a pilot study by Buccino et al. (2011) in a small cohort of PD patients reported that AOT might improve autonomy in the ADLs, thus representing a promising rehabilitative tool also in this view.

Therefore, the first aim of the present randomized controlled trial was to confirm the benefits of AOT treatment in the ADLs of the patients with PDD, using instruments specifically oriented toward the assessment of ADLs.

A second aim was to test whether the observation of actions could have any effect also on the naming of the actions themselves. This research question arises by the observation that PD patients perform worse than healthy controls in action naming. This could be explained by the evidence that the neural connections between basal ganglia, which are typically impaired in PD, and several cortical areas have a crucial role in integrating motor-semantic information in action-related words (Cotelli et al., 2018).

As the observation of actions pertaining to the human motor repertoire was found to be an effective rehabilitation approach for verb recovery in aphasic patients (Marangolo et al., 2012; Murteira and Nickels, 2020), we thought it would be interesting to investigate whether a similar effect could be found also in PDD patients.

## 2 Materials and methods

### 2.1 Patients

Twenty PDD patients were enrolled in the present study at the Unit of Neurology of the National Institute of Health and Science on Aging (Istituto Nazionale di Ricovero e Cura dell’Anziano, Istituto a carattere scientifico, INRCA-IRCCS) Hospital in Ancona, Italy.

The inclusion criteria were all the following:

- A PD diagnosis made according to Movement Disorders Society (MDS) clinical diagnostic criteria (Postuma et al., 2015) at mild–moderate PD stage (Hoehn & Yahr’s stage scale: 2–3).
- Diagnosis of dementia associated with PD according to Emre et al. (2007) criteria. The neuropsychological battery used to the diagnosis of dementia is reported in Supplementary Table A.
- Mini Mental Parkinson Test (MMPT) score corrected for age and education <25.42 (Costa et al., 2013).
- Need of assistance in instrumental activities of daily living (IADL), i.e., IADL less than 8 (Lawton and Brody, 1969) and in basic activities of daily living (BADLs), i.e., BADL less than 6 (Katz et al., 1970).
- Education level of 5 years or more.
- Presence of a caregiver able to accompany the patient to the hospital and to provide information on the clinical status and treatment of subjects enrolled.
- Ability to sign informed consent.

The exclusion criteria were:

- History of neurological conditions other than PD.
- Presence of deep brain stimulation.
- History of psychiatric diseases such as major depression disorders and schizophrenia.
- Presence of sensory impairments potentially interfering with treatment.
- Presence of major medical conditions (for instance severe peripheral artery disease or relevant orthopedic diseases) potentially affecting motor abilities of patients.

During the study, all patients would continue to follow the prescribed pharmacological therapies and normal physiotherapy.

After recruiting 20 subjects, we proceeded with simple randomization which guarantees the randomness of the draws. Randomness was obtained by extracting numbers from a list (called “sampling list”) in which all the individuals of the population to be studied were present. Considering the small number of subjects, randomized permutation blocks system was used.

Two groups of patients were formed: 10 subjects joined the experimental group (AOT-group) and the other 10 joined the control group (C-group).

All subjects underwent motor, functional and language assessment before and after the treatment.

### 2.2 Outcomes

#### 2.2.1 Primary outcome

- ADLs have been assessed through Alzheimer’s disease Cooperative Study-Activities of Daily Living Scale (ADCs-ADL) (Galasko et al., 1997).

The ADCS-ADL assesses the competence of patients with Alzheimer’s Disease (AD) in basic and instrumental ADLs. It is also recommended in PD (Holden et al., 2016). It was administered by a clinician/researcher as a structured interview with a caregiver. The scale is composed by 23 items, which provides descriptions of level of competence, with the rater selecting the most appropriate option. The dependent variable was the sum of the scores (max 78), with higher scores indicating better functioning.

#### 2.2.2 Secondary outcomes

- Motor skills have been evaluated by Unified Parkinson’s disease Rating Scale (UPDRS-part III) (Antonini et al., 2013). This scale is used to assess the longitudinal course of PD. The part III of the scale evaluates the motor signs of PD. The UPDRS-part III scores can vary between 0 and 128, with higher scores indicating worse performances.
- The language impairment has been evaluated by the subtests of oral verb naming (20 items) of the Analysis of Aphasic Deficit Battery (BADA) (Miceli et al., 1994). The dependent variable was the sum of the items correctly and accurately reported, with higher score indicating a better performance.
- Functional skills have been assessed by the Direct Assessment of Functional Status scale (DAFS) (Zanetti et al., 1998; McDougall et al., 2010). It is a performance-based assessment. The maximum score of the Italian version of DAFS is 86, with higher scores indicating better performance.

### 2.3 Treatment

The AOT-group was submitted to AOT treatment through the vision of a video containing 6 complex ADLs that are listed in Supplementary Table B, always during the “on phase” of illness, i.e., during time when the pharmacological PD therapy with levodopa gives the maximum benefit to the patient.

The subjects were given the video clip of the treatment on DVD or other computer support. The video-clip lasted about 30 min. After the vision, subsequent execution of the treatment actions was provided. The treatment took place once a day, for 5 days a week at home and for 2 days a week at the hospital clinic.

The treatment lasted 4 weeks.

The video was recorded and edited by the AOT-PARK project team, as it is usually done with other similar studies (Monticone et al., 2015; Pelosin et al., 2010). The followings steps were made:

- the actions were shown by an actor suffering from PD, since the “mirror” effect seems to be more effective if the actions are carried out by subjects suffering from the same illness (Poliakoff, 2013);
- the actions were accompanied by a voice-over voice that explained the actions step by step in a timely and clear manner, as a multisensory stimulus may be more effective than an exclusively visual stimulus (Schiavio and Altenmüller, 2015);
- the actions were shown from three different angles, as the effect is greater if the action can be viewed from several points of view (Humphries et al., 2016).

The C-group was subjected to a video-clip regarding semantic information of a geographical-naturalistic type with no motor content, always during the “on phase” of illness.

The subjects were provided with the above video-clip, lasting about 30 min, on DVD or other computer support and the vision took place at home once a day during the “on phase” for 4 weeks.

In order to ensure adequate adherence to the treatment, the caregivers of both groups were involved.

### 2.4 Statistical analysis

Data were expressed by mean and standard deviation for continuous variables and by frequency and percentages for categorical ones.

Comparisons between groups were performed by t-test for continuous variables and by chi-square test for categorical ones.

Afterwards, we compared all the variables of interest in the time (baseline/end of study) using paired samples t test, dividing by groups.

Statistical significance was set at *p* < 0.05.

All analyses were performed using IBM SPSS Statistic for Windows, Version 24.0 (IBM Corp., Armonk, NY, USA).

## 3 Results

Demographic and clinical characteristics of 20 patients at baseline divided by group (C-group; AOT-group) are reported in Tables 1, 2.

**Table 1:** Demographic and clinical characteristics of study patients at baseline divided by group.

	All (n = 20)	Control group (*n* = 10)	Case group (*n* = 10)	*P*
Age, years	75.7 (4.7)	74.9 (4.4)	76.6 (5.1)	0.435
Gender, female	7 (35.0%)	4 (40.0%)	3 (30.0%)	0.639
Education, years	7.5 (3.0)	7.7 (3.4)	7.2 (2.7)	0.723
MMPT	21.4 (2.8)	20.8 (3.5)	22.0 (2.2)	0.391
MMSE	23.5 (2.6)	23.2 (2.9)	23.9 (2.4)	0.578

Data are mean (SD) or number of cases (percentage).

**Table 2:** Descriptive statistics at baseline for all measures of interest by group (AOT-group vs. C-group).

		All (*n* = 20)	C-group (*n* = 10)	AOT-group (*n* = 10)	*p*
UPDRS		23 (5.1)	21.9 (5.8)	24.2 (4.2)	0.328
ADCS-ADL		46 (13.2)	47.7 (13.1)	44.3 (13.7)	0.577
BADA		18.4 (3.7)	17.6 (4.0)	19.2 (3.3)	0.346
DAFs		64.9 (12.0)	64.6 (13.8)	65.3 (10.7)	0.901

Data are mean (SD).

The two groups did not differ in terms of age, gender, years of education, MMPT, MMSE and also for measures of interest as UPDRS, ADCS-ADL, BADA, DAFs.

From baseline to the end of treatment after 4 weeks, in AOT-group there were significant improvements for ADCS-ADL (from 44.3 to 50.4; *p* = 0.001), DAFS (from 65.3 to 72.9; *p* = 0.005) and Subtest Verb Naming of BADA (from 19.2 to 21.5; *p* = 0.011), while with respect to UPDRS part III no significant change was found (see Table 3).

**Table 3:** Time comparisons for all measures of interest divided by groups.

	AOT-group (Baseline)	AOT-group (Follow-up)	*p*	C-group (Baseline)	C-group (Follow-up)	*p*
	(*n* = 10)	(*n* = 10)		(*n* = 10)	(*n* = 10)	
UPDRS	24.2 (4.2)	23.6 (4.2)	0.584	21.9 (5.9)	23.3 (7.0)	0.122
ADCS-ADL	44.3 (13.7)	50.4 (13.0)	0.001	47.7 (13.1)	46.1 (12.9)	0.057
BADA	19.2 (3.3)	21.5 (3.1)	0.011	17.6 (4.0)	16.9 (2.3)	0.448
DAFs	65.3 (10.7)	72.9 (5.3)	0.005	64.6 (13.8)	64.6 (13.4)	0.999

Data are mean (SD).

The C-group did not show any significant improvement for all the measures of interest.

## 4 Discussion

The results of this preliminary study suggest potential insights concerning effective strategies in the context of the rehabilitation of PDD persons. The first aims of the present study were to assess the benefits of AOT treatment in the ADLs; the secondary aims were to assess the efficacy in the verb naming and in motor of the PDD patients at mild–moderate stage.

We found that AOT treatment may play a role in the recovery of ADLs of PDD patients and that it improves their performances on action naming.

Similar results were reported by Buccino et al. (2011), who experimented AOT in a small number of PD patients (7 cases and 8 controls). However, this study had relevant limitations in the evaluation tools (Unified Parkinson’s disease Rating Scale-UPDRS and Functional Independence Measures-FIM), which are not specifically oriented toward ADL. Conversely, we carried out an in-depth assessment of ADLs by the ADCS-ADL scale, which is an informant-reported scale specifically designed to assess the competence of patients in basic and instrumental ADLs, and by DAFS, which is a direct assessment of ADLs.

It is therefore reasonable to think that AOT is effective because it targets the Mirror Neuron System and activates the central representation of the behavior, which enhances learning and neural traces of motor actions. In humans the observation of action stimulates neural circuits similar to those involved in action planning and execution and some findings demonstrated that PD patients can still be engaged in action observation to some extent (Poliakoff, 2013).

Furthermore, the same basic principles relevant to enhance motor performance may also be efficacious to improve language functions. Some papers (Marangolo et al., 2010, 2012) reported improvement in verb retrieval only by observing video-clips of human actions in patients with aphasia and confirm that AOT can be a useful strategy to promote long lasting recovery in verb production.

We found similar results in our PDD patients. They presented significant improvement in the verb naming task of the BADA test after the AOT.

In agreement with the embodied cognition viewpoint, it has been suggested that gesture and speech are strongly connected to the same conceptual representation and that gestures are more likely encoded in a multimodal representation including sensory-motor features (Gallese and Lakoff, 2005). If the word is grounded in sensory-motor features, such as a verb, the actual execution and observation of gestures may enhance the activation of the sensory-motor representations in the semantic system and facilitate its retrieval. Therefore, the observation of an action carried out by others is sufficient to activate in the semantic system its corresponding sensory-motor representation, which serves as an input to facilitate retrieval at the lexical level.

Also in this case it is reasonable to think that this effect is due to the involvement of the Mirror Neuron System, which is active both when an action is performed and when it is observed.

Faced to positive results on linguistic abilities and functional competence, we did not find a clear motor efficacy of AOT: indeed, differently from the systematic review by Giannakopoulos et al. (2022), our study did not show any statistically significant effect of this rehabilitation approach on UPDRS part III score, but just a trend toward an improvement in AOT group and a trend toward a worsening in C-group. This suggests that the replication of our study in a larger cohort of PD patients might show a significant efficacy of the here proposed AOT approach also in terms of motor improvement. Furthermore, it should be taken into account that the selection of videos representing mainly fine motor skills in our investigation was typically oriented to try to produce an effect on functional competence (which is the primary outcome of the study) and not on strictly understood motor abilities as assessed by UPDRS part III (which encompasses both fine and gross motors skills). This is a crucial point, because, as specified in the above-mentioned systematic review, the characteristics of stimulus play a relevant role on AOT motor efficacy and in literature there is a significant heterogeneity of intervention features among studies focused on AOT. In other terms, a different selection of motor videos could produce different effects compared to those found in the present investigation.

In summary, our findings provide preliminary evidence on the efficacy of AOT in the rehabilitation of ADLs and language in PDD persons. However, it is important to acknowledge that our study presents some significant limitations.

First of all, given the limited number of patients, the results cannot be considered conclusive and deserve further investigation.

The lack of follow-up evaluation after the end of the treatment is another relevant limitation that might be addressed in further studies.

A third aspect that should be considered is the fact that in our study, like in another analogous studies on AOT (Buccino et al., 2011; Pelosin et al., 2018) the control group underwent a naturalistic video and not a video with motor contents without rehabilitation approach. This aspect is both a strength of the study and a potential source of bias. Indeed, on one hand, our design investigation allowed to make a precise comparison between the rehabilitative effects of visive stimuli acting on neuron mirrors and visive stimuli without any effect of neuron mirrors, thus furnishing an estimation of the validity of neuron mirror paradigm in the context of rehabilitation of PDD patients (it would be impossible if the C-group had been subjected to motor video); but, on other hand, this implies that our study could be affected by a potential overestimation of AOT efficacy due to placebo effect.

Furthermore, the fact that we focused our attention only on PDD patients with H&Y 2–3 clearly limits the generalizability of the results.

Finally, some studies highlighted some factors potentially influencing ADLs, such as gender (Sperens et al., 2020) and the presence of behavioral disorders (Chen et al., 2022), that were not evaluated in the present investigation. Therefore, further studies should take into account these factors that might influence patient outcome.

Taking into account all these relevant limitations, our study should be considered only an exploratory investigation and not a definite demonstration of AOT efficacy in PDD context. Further larger studies indeed will be necessary to confirm these preliminary results.

In conclusion, we believe that these new promising findings might contribute to open future directions for the research about new rehabilitation approaches in PD. We hope that future studies will explore these research directions in order to verify the validity of AOT approach in the improvement of functional competence and linguistic abilities of PDD subjects.

## Data Availability

The raw data supporting the conclusions of this article will be made available by the authors, without undue reservation.
