# Supplementary material for: Action observation treatment may improve daily living activities and verb recovery in Parkinson’s disease-dementia: findings from a preliminary randomized controlled trial
Source: Front Aging Neurosci. 2024 Dec 5;16:1488881. doi: 10.3389/fnagi.2024.1488881 (PMC11655465; doi:10.3389/fnagi.2024.1488881)
Supplement: Supplementary file 1 [file Table_1.docx]

Supplementary Materials

**Table A.** Extensive neuropsychological assessment

| Neuropsychological dimensions | Assessment tools |
| --- | --- |
| Attention and executive functions | Trail Making Test A, TMT-A and Trail Making Test, TMT-B (Amodio et al., 2002)  Stroop Test (Caffarra et al., 2002)  Weigl’s Sorting Test (Laiacona et al., 2000)  Multiple Features Target Test (MFTC) time; accuracy; error (Gainotti et al., 2001)  Frontal Assessment Battery (FAB) (Apollonio et al., 2005) |
| Memory | Digit span (Monaco et al., 2013) |
|  | Rey Auditory Verbal Learning Test-RAVLT, immediate and delayed recall (Carlesimo et al., 1996) |
|  | Rey-Osterrieth Complex Figure B: immediate and delayed recall  (Luzzi et al., 2011) |
| Language | Fluency for semantic categories (Costa et al., 2014) |
|  | Phonemic Fluency (FAS) (Costa et al., 2014) |
|  | Noun Naming (CAGI) (Catricalà et al., 2013) |
| Visual constructional ability | Rey-Osterrieth Complex Figure B copy (Luzzi et al., 2011) |

**Table B**. ADLs

| *Treatment actions* |
| --- |
| Pick up, put on and fasten a jacket with zip |
| Take and read a magazine |
| Take the necessary amount and prepare a soluble chocolate |
| Set the table |
| Draw a star |
| Cut a piece of pong on a plate with knife |

**REFERENCES**

Amodio, P., Wenin, H., Del Piccolo, F., Mapelli, D., Montagnese, S., Pellegrini, A, et al. (2002). Variability of trail making test, symbol digit test and line trait test in normal people. A normative study taking into account age-dependent decline and so-ciobiological variables. *Aging Clin. Exp. Res.* **14**, 117-131. doi: 10.1007/BF03324425

**Caffarra, P., Vezzadini, G., Dieci, F., Zonato, F., and Venneri, A., (2002). Una versione abbreviata del test di Stroop: Dati normativi nella popolazione italiana. *Riv. Neurol.*, 12, 111-115**.

Laiacona, M., Inzaghi, M.G., De Tanti, A., and Capitani, E., (2000**).** Wisconsin card sorting test: A new global score, with Italian norms, and its relationship with the Weigl sorting test. *Neu-rol. Sci.*, **21**, 279-291. doi: 10.1007/s100720070065

Gainotti, G., Marra, C., and Villa, G. (2001). A double dissociation between accuracy and time of execution on attentional tasks in Alzheimer’s disease and multi-infarct dementia. *Brain*, **124**, 731-738. doi: 10.1093/brain/124.4.731

Appollonio, I., Leone, M., Isella, V., Piamarta, F., Consoli, T., Villa, M.L., et al. (2005). The Frontal Assesment Battery (FAB): Normative values in an Ital-ian population sample. *Neurol. Sci.*, 26, 108-116. doi: 10.1007/s10072-005-0443-4

Monaco, M., Costa, A., Caltagirone, C., and Carlesimo, G.A. (2013). Forward and backward span for verbal and visuo-spatial data: Standardization and normative data from an Italian adult population. *Neurol. Sci.*, 34, 749-754. doi: 10.1007/s10072-012-1130-x

Carlesimo, G.A. Caltagirone, C., and Gainotti, G. (1996). The Mental Deterioration Battery: Normative data, diagnostic reliability and qualitative analyses of cognitive impairment. The Group for the Standardization of the Mental Deterioration Battery. *Eur. Neurol.*, 36, 378-384. doi: 10.1159/000117297

Luzzi, S., Pesallaccia, M., Fabi, K., Muti, M., Viticchi, G., Provinciali, L., et al. (2011). Non-verbal memory measured by Rey-Osterrieth Complex Figure B: Normative data. *Neurol. Sci*., 32, 1081-1089. doi: 10.1007/s10072-011-0641-1

Costa, A., Bagoj, E., Monaco, M., Zabberoni, S.,De Rosa, S., Papantonio, A.M., et al. (2014). Standardization and normative data obtained the Italian population for a new verbal fluency instrument, the phonemic/semantic alternate fluency test. *Neurol. Sci.*, 35, 365-372. doi: 10.1007/s10072-013-1520-8

Catricalà, E., Della Rosa, P.A., Ginex, V., Mussetti, Z., Plebani, V., Cappa, S.F., (2013). An Italian battery for the assessment of semantic memory disorders. *Neurol. Sci.*, **34**, 985-993. doi: 10.1007/s10072-012-1181-z
